# Prevalence of depression and associated factors among primary caregivers of adult cancer patients, Sidama region Southern Ethiopia: cross-sectional study

**DOI:** 10.1186/s12912-024-02061-1

**Published:** 2024-06-06

**Authors:** Gulema Demissie, Bargude Balta

**Affiliations:** Hawassa University Comprehensive Specialized Hospital Oncology Center, Hawassa, Ethiopia

**Keywords:** Ethiopia, Caregivers, Cancer, Depression, Prevalence

## Abstract

**Background:**

The effect of cancer diagnosis affects the psychological well-being of the caregivers of cancer patients and results in a risk of psychiatric morbidity. This study aimed to determine the prevalence and associated factors of depression among primary caregivers of adult cancer patients.

**Objective:**

This study aimed to assess the magnitude of depression and associated factors among primary caregivers of adult cancer patients.

**Methodology:**

Hospital-based cross-sectional study was conducted among primary caregivers of adult cancer patients. The convenient sampling method used to recruit caregivers of cancer patients. The data was collected by using the Amharic version patient health questionnaire and analyzed by SPSS version 25. Descriptive statistics were used to describe the prevalence of depression and bivariable and multivariable regression models were used to determine the net effect of each independent variable on depression.

**Results:**

The overall prevalence of depression among adult cancer patient caregivers was 54.1% (95% CI 47.6, 60.6). Household size < 3; (AOR = 4.5, 95% CI: 1.1–13), Monthly income < 600 (AOR = 2.8, 95% CI:2.5–15.9), Caring hours ≥ 9 (AOR = 9, 95% CI:4–21), Burden level ≥ 20 ;(AOR = 10.7, 95% CI:9.3–11.6) were independent factors of depression among primary caregivers of cancer patients.

**Conclusion:**

The results of this study showed a higher prevalence of depressed symptoms among primary caregivers of cancer patients. Long caring hours, small household size, low-income level, and higher burden level were independent factors of caregiver depression, indicating the urgent necessity to investigate and deal with it through interdisciplinary approaches.

## Introduction

Global Burden of Disease estimates for 2018 show that there were 9.6 million cancer deaths and 18.1 million new cases worldwide, respectively [1]. In the United States, the lifetime risk of developing invasive cancer is estimated to be 42% for men and 38% for women [2]. Africa and other low- and middle-income nations accounted for over half of all cancer cases and roughly two-thirds of cancer-related fatalities. In this area, cancer patients and their families experience prolonged depression and have little to no hope [3].

According to estimates, Ethiopia has 64,285 new cases of cancer per year, more than 44,000 fatalities, and a 5.8% overall national mortality rate. There is an 11.3% and 9.4% chance, respectively, of receiving a cancer diagnosis and passing away from the illness [4]. Because cancer affects a patient’s social environment, its effects extend beyond the individual suffering from the disease. There is a chance of psychiatric morbidity as a result of the impact cancer has on the mental health of those who care for cancer patients [2].

Cancer diagnosis and treatment are traumatic experiences that place a heavy load on carers and can elicit emotional reactions like depression and anxiety [5]. Although they are valuable members of the oncology team, carers for cancer patients are frequently disregarded. From diagnosis through treatment and ultimately death, care has an impact. The effects of providing care are still mainly unclear in developing nations. The patient’s spouse, son, daughter, friend, or other close relatives are the most common family members who take on the role of carer and are crucial to the patient’s care and recovery [6, 7].

Cancer causes many disruptions to carers, but the most significant burden is related to their psychological well-being. In the psychological domain of quality of life, the effects manifest as increased depression and difficulty coping with caregiving responsibilities [8–10]. Family carers provide long-term care for cancer patients; however, they are not adequately prepared, informed, or supported to carry out these crucial roles [11]. Research showed that carers’ levels of depression are higher and do not gradually subside compared to those of the cancer patients they are caring for, with prevalence rates ranging from 3 to 82% [12–14]. The demographic characteristics of the patients and their caregivers, length of hours of stay caring for the cancer patients, clinical variables of the patient, and caregivers’ burden level have been considered to play a role in the development of depression in caregivers of cancer patients [7, 8, 13–27].

Research indicated that early detection and treatment of depression in carers may lead to better physical and mental health, a higher quality of life, a reduction in suffering, and an enhanced ability to care for cancer patients when provided with psychoeducation, skill development, and therapeutic counselling [13, 28, 29]. Research indicates that in Ethiopia, the prevalence of depression is lower in the general population than in chronic illnesses [30]. Studies on cancer patients’ carers are scarce, though. and the baseline for future research would be this study. Thus, the purpose of this study was to evaluate the prevalence of depression and related variables among primary carers of adult cancer patients who visited the South Ethiopian HUCSH oncology unit in 2019.

## Methods and materials

### Study area and study period

This cross-sectional study was conducted in Hawassa City University Hospital, which is about 300 km away from the capital city of Ethiopia Addis Ababa to the south. The Hospital is the largest in the Southern Nation of the Ethiopian region. The hospital started all cancer treatment starting from 2017 GC. Currently HUCSH cancer center has two senior clinical oncologists, two MSc in clinical oncology nurses, four trained general practitioners, one palliative care physician 12 trained nurses, three clinical pharmacists, two laboratory technologists, and supportive staff. HUCSH Cancer Centre has 18 functional beds and the Centre is on preparation to start radiation therapy. with three months average outpatient visit is 250 per month. Colorectal, breast, cervical, and sarcomas are the commonly seen cases at the Oncology center. The study was conducted from March to June 2019.

### The study population

The source population was all cancer patients’ caregivers who attended the oncology unit. The caregivers aged > 18 years, who can understand Amharic, and Caregivers with sufficient general conditions to interview were included in the study. Professional or paid caregivers, caregivers who stayed with the patient for less than two weeks, caregivers with a previous history of depression, and caregivers of newly diagnosed cancer patients with less than 2 weeks were excluded from the study.

### Sample size and sampling procedure

The Sample size was determined by calculating using a single population proportion.

Sample size calculation formula n= (z_α/2_)^2^pq/d^2^.

Using a confidence level of 95% (α = 0.05; z_α/2_= 1.96 and a maximum permissible error of 5% and since there is no similar study found in Ethiopia *p* = 50% was used. The result of the calculation was *n* = 384 and considering 10% for missing data and for non-response rate, the final sample size became 422. The final caregivers who attended cancer patients during the survey period were 238. So, the sample size for the study was all caregivers who attended cancer patients during data collection. No specific sampling procedures were employed since all caregivers attended cancer patients were included in the study. For each patient, we selected one caregiver based on cancer patients’ recommendations and inclusion criteria.

### Operational definition

**Depression in caregivers**- depression in caregivers was measured by using the PHQ-9 depression assessment tool. Those caregivers who scored greater than or equal to 10 were taken as depressed [31, 32].

**Primary caregivers**- Family members include fathers, mothers, sisters, brothers, daughters, sons, uncles, aunts, grandfathers, partners, sons or daughters-in-law, and other blood relations. They should be the ones who stay with the patient for two weeks or more and are not compensated for their care [28].

**Caregiver burden** -was assessed with the Zarit Burden Interview (ZBI). ZBI consists of 24 questions scored on a 5-point Likert scale from 1 (strongly disagree) to 5 (strongly agree), covering five domains evaluating the caregivers’ reactions to caring at the time of the survey. The higher ZBI scores indicate the greater the burden. No burden at scores of less than 10, mild burden at 11 to 20, and severe burden at ≥ 20 [33].

### Data collection procedures and quality assurance

The participants were identified by asking the patient who was their caregivers. Data were collected by using structured questionnaires, a patient health questionnaire (PHQ-9), and a Zarit Burden Interview (ZBI). PHQ-9 was validated to be used for screening and diagnosis of depression in caregivers [31]; it was also validated in Ethiopia (Amharic version) with a reliability test of Cronbach’s alpha 0.89 [33]. ZBI was used to assess caregivers’ burden and translated into Amharic language and back to English to check its consistency by fluent English and Amharic speakers [34]. The clinical variables of patients were collected from patients’ health care profiles by prepared format based on the clinical variables that need to be collected. *A pilot study* was done 2 weeks before data collection at a private Yanet Higher Specialized clinic chemotherapy center.

In this study, a pilot test was conducted on 30 participant caregivers during the pre-test. Structural validity was validated by face validity and internal consistency (reliability) was checked by Cronbach’s alpha and it was α = 0.89. Quality was assured by conducting a pre-test and giving training for the data collectors and supervisors before the actual data collection. Appropriate modifications were made after viewing the pre-test result and overall supervision was made by the principal investigator. The pilot study indicated that the participants needed to be interviewed privately and the necessity of adding some variables. The interviews took 10–15 min for quantitative.

### Statistical analysis

All filled checklists/questionnaires were checked for completeness and consistency, cleaned manually, and entered into Statistical Package for Social Science (SPSS) Windows version 25. Descriptive statistics were used for the socio-demographics, caregivers’ related burden, and clinical variables of the patient and depression. The variables with a *p*-value less than 0.25 in bivariate logistic regression were interred into multivariate logistic regression. The variable with a *p*-value less than 0.05 in multivariate logistic regression was declared as a predictive associative factor of depression of primary caregivers among adult cancer patients attending the HUCSH oncology unit.

## Results

### Socio-demographic characteristics of the cancer patients’ caregivers

A total of 238 study participants were enrolled in the study 231 responded with a response rate of 97%. The mean age of the caregivers was 35 years. About 139 (60%) of the respondents of cancer patients’ caregivers were female whereas, 155 (67.1%) of the caregivers were male. The majority 203 (87.4%) of the participant patients were married. The mean, median, and maximum household sizes of the caregivers were 5, 5, and 16 respectively. 78.4% of caregivers were employed, more than 90% of caregiver’s education level was primary and above, 61.5% of the caregivers live with their patients and only 2.2% of the caregivers did not know the diagnosis of their relatives’ disease type (Table 1).

**Table 1:** Socio-demographic characteristics of caregivers attending cancer patients at HUCSH oncology unit, Hawassa, 2019(*n* = 231)

Variables		Frequency	Percent
Age groups	15–24	10	4.3
	25–34	61	26.4
	35–44	91	39.4
	45–54	46	19.9
	55–64	15	6.5
	> 65	8	3.5
Gender	Male	155	67.1
	Female	76	32.9
Employment	Employed	181	78.4
	Unemployed	50	21.6
Marital status	Married	173	74.9
	Not married	58	25.1
Monthly	< 600	69	29.9
Income	601–1650	37	16
	1651–3200	47	20.3
	3201–5250	47	20.3
	5251–7800	16	6.9
	> 7801	15	6.5
Residence	Urban	145	62.8
	Rural	86	37.2
Educational	No education	22	9.5
level	Primary	47	20.35
	Secondary	83	35.9
	Above secondary	79	34.2
Household	< 3	63	27.3
size	4–5	54	23.4
	> 6	114	49.4
Caring hours	< 4	96	41.6
	5–8	59	25.5
	> 9	76	32.9

### Caregiver characteristics

**Fig. 1:**
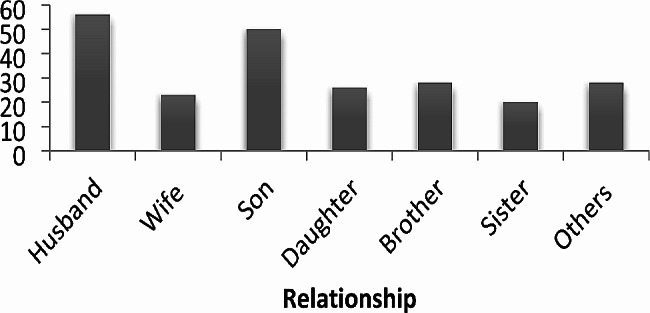
The relationship of primary caregivers with adult cancer patients attending the oncology unit, 2019(*n* = 231) Note: Others includes- father, mother, close friends and volunteer supporters with less frequency observed

Regarding the type of caregivers of cancer patients, the husband and son of the patient were the predominant numbers of caregivers (Fig. 1).

### Clinical characteristics of cancer patients

**Fig. 2:**
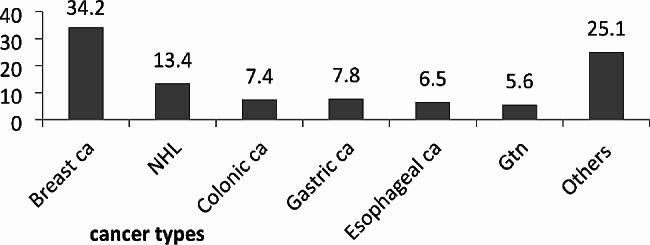
Distribution of adult cancer types of the cancer patients of the study attending the oncology unit, 2019(*n* = 231)

Breast cancer accounts for a large proportion of distribution in treatment centers (34.2%) followed by GI (15.2%) and NHL (13.4%) cancers and others were classified under 14 different less frequent cancer types categorized under other types accounting for 25.1%. About 213(92.2%) of patients were under treatment among these 118(51.1%) chemotherapies (either on daycare or inpatient). The 7(3.03%) of cancer patients were capable only of limited self-care and confined to bed or chair for more than 50% of working hours (with performance status ECOG 3). The majority of the patients were in the advanced stage; presented with stage three 89(38.5%) and stage four 81 (35.1%). Around 25(10.8%) cancer patients have co-morbidity (Table 2), (Fig. 2).

**Table 2:** The clinical characteristics of the adult cancer patients attending HUCSH oncology unit, Hawassa, 2019 (*n* = 231). Variables Frequency Percent

Physical	ECOG I	176	76.2
performance status	ECOG II	48	20.8
	ECOG III	7	3
Cancer stage	I	6	2.6
	II	55	23.8
	III	89	38.5
	IV	81	35.1
Duration of	< 3 months	43	20.2
treatment	≥ 3 months	170	79.8
Clinical symptoms	Present	150	64.9
	Absent	81	35.1
Treatment started	Yes	213	92.2
	No	18	7.8
Type of treatment	Chemotherapy	118	51.1
modalities	Surgery alone	32	13.9
	Chemo plus surgery	60	25.9
	Hormonal	21	9.1
Co-morbidity	Present	21	9.1
	Absent	210	90.9
Type of comorbidity	Anemia	13	61.9
	HTN	5	23.8
	DM	3	14.3

ECOG-Eastern Cooperative Oncology Group, HTN-Hypertension, DM- Diabetes Mellitus

### Depression and burden level of caregivers

**Fig. 3:**
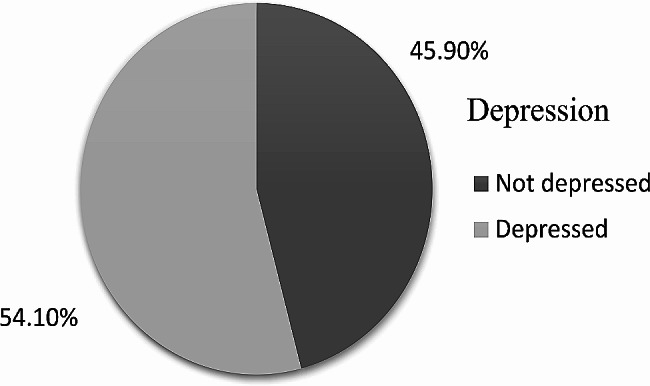
Depression of primary caregivers of adult cancer patients attending oncology unit, 2019(*n* = 231)

The mean caregivers’ depression score was 9.5 with a standard deviation of 5.7. A 6(2.6) % of caregivers had severe depression. The mean burden score of the respondent caregivers was 15 and the median score was 13. The mean hours of the caregivers who stay with and give care for the patients were 9.36 (SD 8.41) and the median hour is 6. 51 out of 231 caregivers respond that give care for their relatives 24 h per day. One-third (32.9%) of the caregivers spent their time caring for their relatives more than the mean hours of caring. The majority 96(41.6%) has a burden were 10–20 and the majority 76(32.9%) have a depression range of 10–14 (Table 3), (Fig. 3).

**Table 3:** The depression and burden level of primary caregivers attending HUCSH oncology unit, Hawassa, 2019 (*n* = 231)

	Score	Frequency	Percent
Depression level	0–4	50	21.6
	5–9	56	24.2
	10–14	76	32.9
	15–19	43	18.6
	20–27	6	2.6
Burden level	0–10	81	35.1
	10–20	96	41.6
	> 20	54	23.4
Caring hours	< 4	96	41.6
	5–8	59	25.5
	> 9	76	32.9

### Bivariate and multivariate analysis of factors associated with depression

To identify the independent factors of depression among caregivers of cancer patients attending HUCSH, bivariate and multivariate regression models were used. The bivariate regression model age, household size, monthly income, caring hours, and burden level showed a significant association with caregiver depression (Table 4). After multivariate analysis, four baseline factors were identified: household < 3; (AOR = 4.5, 95% CI: 1.1–13), Monthly income < 600 (AOR = 2.8, 95% CI:2.5–15.9), Caring hours ≥ 9 (AOR = 9, 95% CI:4–21), Burden level ≥ 2020 ;(AOR = 10.7, 95% CI:9.3–11.6) are independent factors of depression among primary caregivers of cancer patients (Table 4).

**Table 4:** Bivariate and multivariate analysis of factors associated with depression of primary caregivers of adult cancer patients attending HUCSH oncology unit, Hawassa, 2019 (*n* = 231)

Characteristics	Depression		Crude odd ratio (95% CI)	Adjusted odd ratio (95% CI)
	No	Yes		
**Age**				
< 19	7(6.6)	3(2.4)	0.19(0.04, 0.95) *	0.15(0.01,1.77)
20–29	32(30.2)	29(23.2)	0.40(0.14, 1.10)	2.29(0.33,15.70)
30–39	40(37.7)	51(40.8)	0.56(0.21, 1.49)	0.71(0.17,3.04)
40–49	20(18.9)	26(20.6)	0.57(0.20, 1.65)	0.22(0.05,0.95) *
> 50	2(6.6)	16(12.8)	1	1
**Household size**				
< 3	34(32.1)	80(64)	8.24(4.02, 16.87) ***	4.5(1.1,13) ***
4–5	23(21.7)	31(23.8)	4.72(2.12, 10.52) ***	2.6(3.3,12)
> 6	49(46.2)	14(11.2)	1	1
**Monthly income**				
Monthly income < 600	30(28.3)	39(31.2)	5.2(1.35, 20.09) *	2.8(2.5,15.9) **
600–1650	11 [11]	26(20.8)	9.46(2.22, 40.24) **	3.9(4.2,24)
1651–3200	19(-17.9)	28(22.4)	5.9(1.46, 23.73)	6.2(0.7.2,14)
3201–5250	26(24.5)	21(16.8)	3.2(0.81, 12.97)	4.7(0.8,23.4)
5251–7800	8(7.5)	8(6.4)	4(0.81, 19.82)	10(0.2,79)
> 7801	12(11.3)	3(2.4)	1	1
**Caring hours**				
Caring hours < 4	66(62.3)	30 [25]	1	1
5–8	26(24.5)	33(26.4)	2.8(1.43, 5.46) **	1.9(0.9,4.3)
≥ 9	14(13.2)	62(49.6)	9.7(4.73, 20.07) ***	9(4,21) ***
**Burden level**				
0–10	64(60.4)	17(13.6)	1	1
10–20	37(34.9)	59(47.2)	6(3.06, 11.8) ***	5.6(0.9,12)
≥ 20	5 (4.5)	49 (39.2)	36.9(13,44.3) ***	10.7(9.3,11.6) ***

1-Reference. * Significant at *p*-value 0.05. ** Significant at *p*-value 0.01. *** Significant at *p*-value 0.001

## Discussion

This study determined the prevalence and associated factors of depression among adult cancer patients’ primary caregivers in the oncology treatment center at Hawassa University Comprehensive Specialized Hospital. The overall prevalence of depression was found to be 54.1% with (95% CI:47.6–60.6). The prevalence of depression was found to be higher than the study finding from U.S.A 6% [6], Uganda 26% [8], Malaysia 29.4% [36], Germany 26% [37], and United Kingdom 3% [38]. The higher prevalence of depression in the current study setting may be due to a comprehensive depression management program alongside cancer management or it could be due to geographical, environmental, lifestyle, and genetic variation. The current study is lower than the findings from South Korea (82.2%) [19] and the UK (58.1%) [39]. These differences may be because of the different sample sizes, service setup, study participants, study period, and sampling technique.

In this study 40–49 age group of caregivers was 78% less likely to develop depression; this finding is similar to the study conducted in Turkey found that younger age groups were more likely to develop depression than older age group [19]. The number of family members of the caregivers was an associated factor of depression among caregivers of cancer patients attending the HUCSH oncology unit. Caregivers with household size < 3 were 4.5 times more likely to be depressed when compared with household size this was congruent with a previous study [40]. This is due to those who have lower household sizes there is limited sharing of burden and a greater chance of longer caring hours which may induce depression in caregivers. So, this may predispose to the development of depression among caregivers.

The monthly income level of caregivers was a significantly associated factor for the development of depression in caregivers. The monthly income of caregivers with better monthly income have a lower chance of getting depression which is similar to previous findings [16, 19]. This may be because they feel mental pain as the reason for the shortage of income due to financial toxicity. After the patient loses his/her wellness, the productivity decreases even some lose their jobs. The caregivers also invest their working hours in the patients and because of this, they may be blamed for not working some are financially punished others are put in a lower position and lower payment. The finding of financial influence on the development of depression among caregivers is similar to an investigation done in Turkey [18].

The daily caring hours were the other associated factor of depression among caregivers of cancer patients. As the caring hour length with patients increases, the development of depression among caregivers increases. Caregivers caring > 9 h per day have a 9 times higher chance of depression. This study’s finding was similar to an investigation done in Athens, Greece, indicating that depression increases with increasing daily caring hours [15]. This may be because of staying longer hours suffering from observing and feeling the hopelessness of the patient, confusion about what to do when the patient suffers from side effects and disease conditions, and sometimes not knowing what to prepare for eating, what is preferable, how to talk to relax him/her.

Burden is a statistically significant associated factor of depression among caregivers of cancer patients. 64.9% of participant caregivers were burdened by their caregiving activities. Caregivers with burden levels 10–20 and < 20 were 5.6 and 10.7 more likely to burden respectively which is Similar to studies conducted in Melbourne Australia, western USA, and Spain [9, 14, 25, 41]. . This may be due to the caregivers not being able to participate in the social function because of caregiving. They couldn’t participate in different weddings, group work, and the like activities. Caregivers were overloaded with their activities. They care for the patient; most of the time generate income, and care for other family members.

This study has certain limitations; First, additional analysis to establish causal inferences was not feasible because of the cross-sectional nature of the study. Second, data was limited to only cancer patients related to the depression of caregivers, making it difficult to extrapolate to other causes of caregivers’ depression. Third the validity of the instruments utilized in this study to measure the outcome variables was not assessed.

## Conclusion

This study aimed to assess the prevalence of depression and its associated factors among caregivers of cancer patients. We found that the prevalence of depression among caregivers of cancer patients was high. Long caring hours, small household size, low income level, and higher burden level were independent factors of caregiver depression, indicating the urgent necessity to investigate and deal with it through interdisciplinary approaches. Caregivers of cancer patients need prevention, screening, and management of depression. We recommend that we establish an organization to work on the psychological wellness of caregivers and depression screening for caregivers.

## Data Availability

All data generated or analysed during this study are included in this article.

## Abbreviations

AOR: Adjusted Odds Ratio
COR: Crude Odds Ratio
CRA: Caregivers Reaction Assessment
ECOG: Eastern Cooperative Oncology Group
PHQ: Patient Health Questionnaire
ZBI: Zarit Burden Interview
